# Application of optimal contribution selection, trait distribution management, and group selection in layer chickens

**DOI:** 10.1186/s12711-025-01029-2

**Published:** 2026-01-21

**Authors:** Anna Wolc, Brian P. Kinghorn, Alexander J. Kinghorn, Jack C.M. Dekkers

**Affiliations:** 1 Department of Animal Science, Iowa State University, Ames, IA 50011- 1178 USA; 2 Hy-Line International, Dallas Center , IA 50063 USA; 3 University of New England, Armidale, Australia; 4 BSK Software, Armidale,, Australia

## Abstract

**Background:**

Optimal Contribution Selection (OCS) aims at maximizing long term response to selection by balancing short term genetic gain and inbreeding rate. In this study, OCS, trait management, and group mating methods were applied to poultry data to evaluate their potential impact. Real data from a White Leghorn line with 7 generations of pedigree and estimated breeding values (EBV) for 18 traits were used to evaluate responses from a single round of selection. It was shown that the current inbreeding rate is low and cannot be substantially reduced without significant loss of genetic gain, unless implemented in parallel with genomics to enable flexibility in population structure.

**Results:**

Allowing flexible mating ratios under OCS resulted in 5.3 to 23.8% more genetic gain plus lower loss of genetic diversity compared to fixed-ratio ocs. However, in the case of multiple males per female, implementation is logistically challenging and requires genotyping of all hatched chicks. Using predicted progeny trait distribution management, a 3.2 g difference in mean EBV for egg weight was obtained for two market-targeted groups, without impact on the predicted progeny mean for the multi-trait index used for selection, or on average parental coancestry, but with a small increase in progeny inbreeding. While maintaining these two egg weight groups, tactical desired gains using trait EBV was used to favourably reduce predicted progeny genetic merit for feed intake from + 0.850 to -0.005 g/day, with little impact on genetic gain for other traits, mean index values, mean parental coancestry, or mean progeny inbreeding. Using pooled semen or multi-sire mating, while accounting for variation in male reproductive success, resulted in only a 0.5% reduction in response in predicted mean progeny index and in small increases in mean parental coancestry (from 0.014 to 0.015) and mean progeny inbreeding (from 0.005 to 0.007).

**Conclusions:**

Evaluating the longer-term impacts of OCS and other methods employed requires multi-generation simulations, ideally starting from the current real data as a base. However, the current approach of using a real implementation scenario is important in decision making for real-life applications. Similar benefits from the selection and mating strategies used here are expected in breeding programs for other species.

## Background

Breeding programs for layers are characterized by intense selection and relatively short generation intervals, in comparison to other livestock systems. This poses a risk of high inbreeding rates, which is partially mitigated by lack of effective methods for freezing semen (inability to move semen across locations prevents extensive use of a few superior males) and maintaining relatively large effective population size. Traditionally, mating took place in fixed groups with a 1:6 to 1:10 male to female ratio, which allowed full pedigree reconstruction by keeping track of which hen laid each egg. Implementation of genomic selection has opened several opportunities to enhance layer breeding programs, from increasing accuracy of estimated breeding values (EBV), through further reductions in generation intervals, and the possibility of parentage assignment, enabling use of pooled semen or group mating, i.e. allowing multiple males to mate with a group of females, as shown by simulations [1] and real data [2].

Optimal Contribution Selection (OCS) was developed to maximize genetic gain while constraining inbreeding rate [3]. As an example, under OCS a very high indexing male might be allocated to more females than would be dictated by conventional wisdom in order to exploit its high genetic merit. The potentially damaging effects of this on genetic diversity are compensated for by the OCS method selecting other stock of appropriately lower coancestry with the rest of the selected population. This results in meeting target outcomes for parental coancestry (i.e. the mean relationship among all parents to be mated, weighted by number of matings per animal), while maximizing the mean genetic merit of the selected animals, or some function of mean genetic merit and parental coancestry. Of course, OCS cannot exploit such opportunities when the male-female mating ratio is fixed, although use of OCS under a fixed mating ratio is still expected to bring some benefit, as OCS will optimize contributions at the levels of grandparents or of other definitions of lineage. Extension to the OCS method would be required when non-additive effects become important, wherever the outcome for a component is not the simple average to two parental values. A topical example is when Mendelian segregation variation can be predicted as a function of interactions between parental genetic markers in their impact on segregation variance. This gives additional opportunities to manage genetic variation for increased long-term genetic gains (e.g [4]).

Theoretical and simulation studies [5] suggest that OCS can benefit layer breeding programs in maintaining long-term genetic gains. Stronger impact was observed in scenarios with more intense selection. In order for OCS to handle large population sizes and deep pedigrees or large genomic relationship matrices that are characteristic for poultry breeding programs, efficient algorithms have been developed [6–10]. Because of the need to balance genetic gain and inbreeding rate, for which there’s no objective optimum, the results of OCS are often presented as a gain-diversity frontier (Pareto front) of possible outcomes for varying importance attributed to these two factors, as applied through weightings or constraints.

The focus of OCS is on selection of males and females for breeding and optimizing their contributions to the next generation, but does not cover mate allocation. While OCS has a major role to play in balancing genetic gains with maintenance of genetic diversity, in some circumstances, appropriate attention to mate allocation can further improve long-term gain and diversity outcomes [4, 9].

The program MateSel (www.matesel.com) implements OCS and also allows for additional features that may be of relevance to poultry breeders, such as group mating and trait management. The latter includes desired gains and managing trait distributions, such as restricting extreme EBVs in parents or predicted progeny, compensatory mating, and assortative mating groups to supply different market requirements for particular traits and genetic markers while maintaining a single population.

The objective of this study was to evaluate the impact of OCS, trait management, and group mating methods, as implemented in the program MateSel, on genetic gain and inbreeding rate, using real data from a commercial layer breeding population. Although details may differ, the principles outlined here can apply to breeding programs for other species also. The use of real data limited the study to a single round of selection but did not need to rely on the many assumptions that are usually inherent to simulations, such as normal distribution of residual effects. Evaluation of multi-generational impacts of the proposed strategies was not undertaken here and would require multi-generation simulation and its associated assumptions. Such studies have been undertaken for different breeding program scenarios (e.g [11–13]).

## Methods

### Dataset

Genomic EBV for 18 traits that were generated for selection in a White Leghorn line in 2023 using single-step multi-trait Best Linear Unbiased Prediction animal models and 7 generations of pedigree were provided by Hy-Line Int. The EBV were assumed to be unbiased and expected progeny means and predicted selection response were computed as the average of the EBV of the parents, assuming no relationship between fertility and the traits under selection. Traits under selection included saleable egg production rate in 4 time periods capturing onset of production, peak production, late production, and persistency (GEGG1-4), age at first egg (AFE), average feed consumption per day (AFC), residual feed intake (RFI), egg mass (EM, average egg weight multiplied by average egg production during feed test), egg weight (EW), breaking strength at equator (BSE) and poles (BSPO), yolk weight (YW), and YW percentage. The egg quality traits were recorded around 35, 65, and 90 weeks of age, which is denoted by 1, 2, and 3 in trait abbreviations. Trait EBV were combined into an index relevant for this breeding population and mostly focused on increasing persistency of production, shell strength, and early egg weight. All birds were kept in individual cages in an optimal, bio-secure environment, with feed and water provided ad-libitum. The analysed generation included 1147 male selection candidates and 3775 female candidates, of which 100 and 600 were selected based on the index in the reference scenario, assuming a fixed 1:6 mating ratio.

## Methods and scenarios used in the analysis

The method used to approach optimal selection and mating solutions was based on [8] and used a mix of stochastic and deterministic optimization strategies competing in a single framework. The method aims to maximize an objective function that includes weights and constraints on component outcomes, including trait means and relationships among selected candidates.

Several functions of the MateSel software and several scenarios potentially relevant to poultry breeders were evaluated:

1. OCS with different male-female mating ratios:
  - A fixed 1 male : 6 female mating ratio, as in the reference scenario. To obtain a range of possible gain and inbreeding values, scenarios with 0, 30, 60, 90 degrees (^o^) for the balance between genetic gain and mean parental coancestry along the gain-diversity Pareto frontier were considered. The Pareto frontier evaluates possible values for response in genetic gain and coancestry for all possible ratios between weights put on index value vs. coancestry. The chosen scenarios range from putting all weight on genetic gain (0^o^) to all weight on maintenance of genetic variation (90^o^), with two intermediate, more balanced approaches (30 and 60 ^o^). In the absence of other objective function components, the 0^o^ scenario is equivalent to performing selection entirely based on the index. Inbreeding rate (as mean parental coancestry based on pedigree), genetic gain in index, progeny inbreeding (based on pedigree), and average EBV of selected individuals for important traits were evaluated.
  - A flexible mating ratio for males, in which each selected male was allowed to be mated with between 2 and 12 females. This scenario was run to obtain the same mean parental coancestry level as achieved at 30^o^ under the fixed 1:6 mating ratio scenario and also at a weighting of 30^o^ under this flexible male mating ratio scenario.
  - A flexible mating ratio for both males (2 to 12 females as per the previous scenario) and females, which were allowed to be mated to 1 to 6 males. Again, the scenario was run to obtain the same mean parental coancestry level as achieved at the 30^o^ standard scenario and at 30^o^ for the new scenario.

(2) Population differentiation by aiming to create the maximum possible divergence in egg weight between two groups by assortative mating, in order to supply markets that have different target egg weights.
(3) Desired gains for feed efficiency, in addition to assortative mating for egg weight, aiming to at least maintain feed efficiency at the current level.
(4) Group mating [14], equivalent to use of pooled semen, by dividing 600 selected females and 100 selected males into 10 equally sized mating groups. If there is variation in male reproductive success within the pools of semen, a correction to predicted parental coancestry must be applied based on a prediction of this variation in male reproductive success within a pool. For this, we used the function described by [15], which predicts the proportion of matings achieved by the *j*^*th*^ most successful sire out of a pool of *n* sires, under a model that uses a two-parameter function of order statistics of selecting *j* out of *n*. We used the default parameters of *m* = 2 and *p* = 3.5, where *m* affects the variability of success and *p* affects the skewness of success. These parameters were derived in [15] using real data on realized numbers of progeny sired by each male in the pool based on genetic marker data, from sheep, beef, and fish. These parameters provided a good fit, both across different numbers of males included in a pool and across these different species.

## Results and discussion

### Impact of OCS on response to selection and inbreeding rate

For the fixed mating ratio, 100 males and 600 females were always selected but flexible scenarios chose different numbers of selected males and females. To maintain the same level of coancestry as for the fixed mating ratio at 30^o^, still close to 100 males were selected in the flexible scenarios but with greater genetic gain, while the 30^o^ flexible scenario favoured more intense selection and slightly greater average parental coancestry (Table 1).

**Table 1:** Response to selection with different optimal contributions scenarios.

	Fixed 1:6 mating ratio, coancestry at					Flexible mating ratio			
	0^o^	30^o^	60^o^	90^o^		2–12 males fixed females coancestry at 30^o^ fixed result	2–12 males 1–6 females coancestry at 30^o^ fixed result	2–12 males fixed females coancestry at 30^o^ flexible result	2–12 males 1–6 females coancestry at 30^o^ flexible result
N selected males	100	100	100	100		89	96	67	78
N selected females	600	600	600	600		600	181	600	168
Mean parental coancestry	0.016 (100%)	0.014 (85.9%)	0.012 (71.8%)	0.012 (70.6%)		0.014 (85.9%)	0.014 (85.9%)	0.016 (98.2%)	0.016 (98.2%)
Mean response in progeny index	1.730 (100%)	1.630 (94.2%)	0.880 (50.9%)	−0.088 (−5.1%)		1.700 (98.3%)	1.985 (114.7%)	1.821 (105.3%)	2.142 (123.8%)
Mean progeny F	0.052	0.005	0.004	0.003		0.005	0.004	0.005	0.005

Numbers of selected parents and expected genetic gain and average parental coancestry for the following optimal contribution selection scenarios: fixed mating ratio of 1 male to 6 females with different weight on gain versus preservation of genetic variance (0, 30, 60, or 90^o^ on the gain-diversity frontier) and flexible mating ratios, with males mated to between 2 and 12 females (2–12 males) and females mated to 1 to 6 males at 30^o^, or average parental coancestry fixed at the value obtained at 30^o^ in the fixed mating scenario. Values in brackets represent % response relative to the 0^o^ scenario

The level of parental coancestry was low in all scenarios (Table 1). For the fixed mating ratio, there was not much difference numerically in the inbreeding rate for different degrees: the fully conservative strategy (90^o^) gave almost 30% lower average parental coancestry than the 0^o^ scenario (0.012 vs. 0.016), but with no genetic gain (progeny mean index response − 0.088 versus 1.730 in Table 1). The 30^o^ scenario was selected for further analysis, which had a 14.1% lower average coancestry and 5.8% lower genetic gain for the index compared to the most aggressive strategy under the fixed mating ratio (0^o^) (Table 1).

**Fig. 1:**
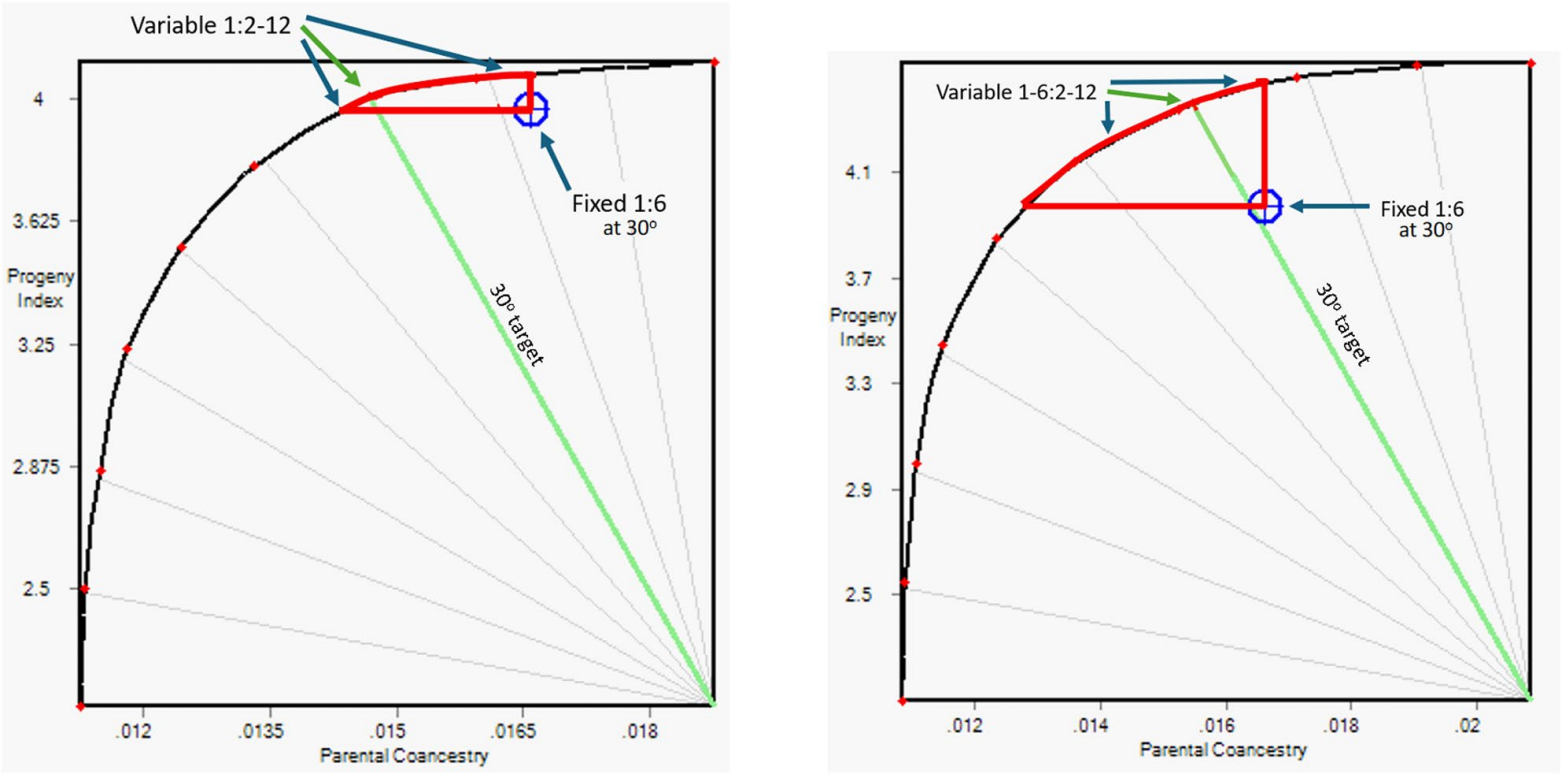
Impact of OCS with a flexible mating ratio on average progeny index and parental coancestry. The curved line is the gain-diversity frontier, representing the maximum mean progeny index (vertical axis) that can be achieved for a given level of mean parental coancestry (horizontal axis). The slant green line shows the minimum permissible degrees for the prevailing analysis (30^o^ in this case). The left-hand graph allows each male to be mated to 2 to 12 females, with each selected female used for at most one mating. The right-hand graph also allows each female to be mated to 1 to 6 males. For each graph, the optimal solution is where the green target degree line intersects with the frontier curve, however any point on the frontier curve can be achieved by changing this target. In both cases the blue icon shows the result for the fixed 1:6 mating ratio scenario at 30^o^. In both graphs, the flexible mating ratio gives greater genetic gain and greater genetic diversity. The red arc on the frontier curve shows the range of improvement in both genetic gain and diversity that can be achieved by moving from a fixed mating ratio to the prevailing variable mating ratio

**Fig. 2:**
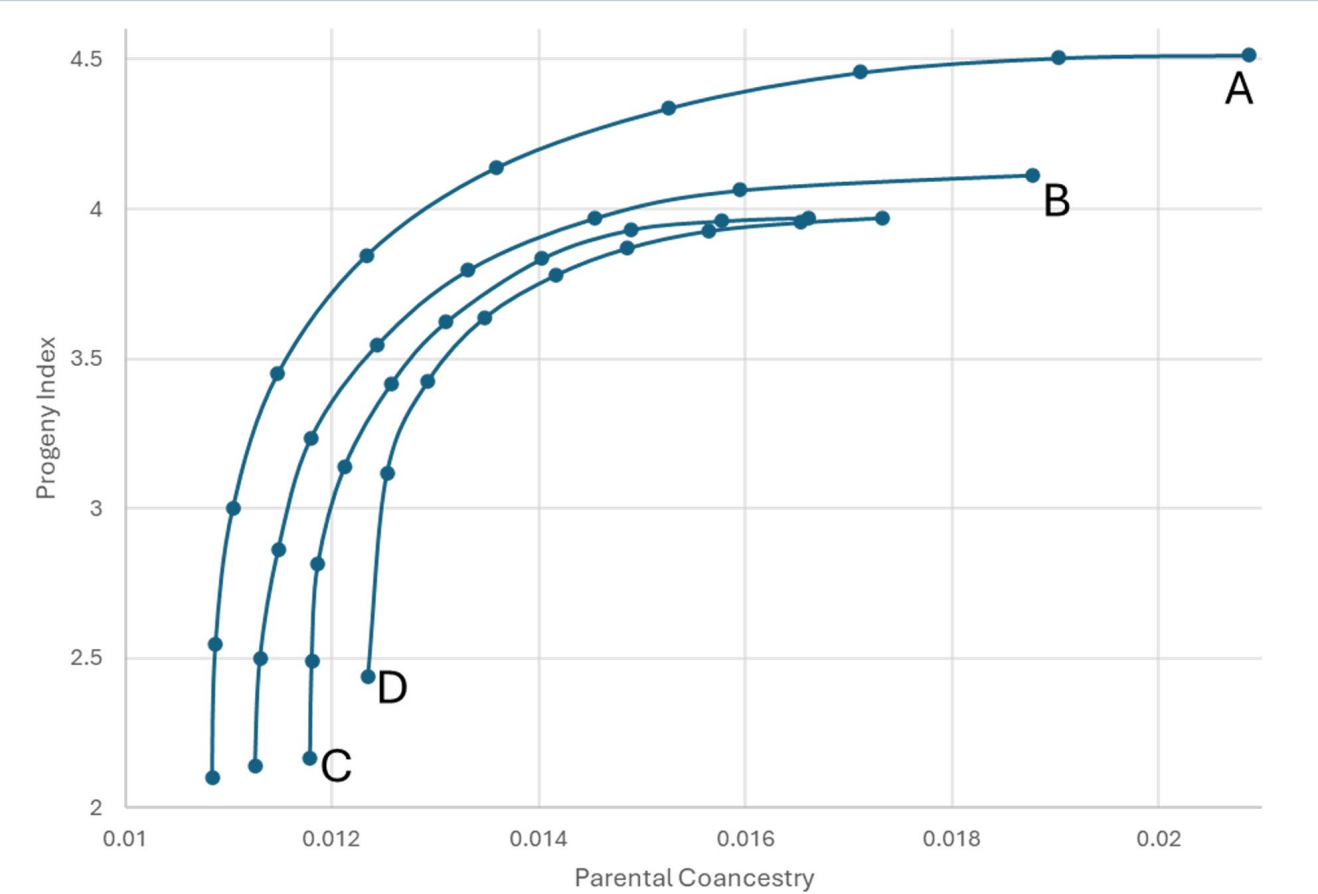
The gain-diversity frontiers from the different analyses. A = flexible male and female mating ratios; B = flexible females per male mating ratio; C = Fixed mating ratio of 1 male to 6 females; D = Mixed mating groups for pooled semen with variation between males in semen fertility

The impact of using flexible mating ratios is shown in Fig. 1. For any one scenario, the extent of possible response in progeny index (for genetic gain) and parental coancestry (for genetic diversity) is shown in a gain-diversity frontier, on which we have chosen a 30^o^ target for illustration. For comparison, the 30^o^ result from the fixed mating ratio analysis was imported into the flexible mating ratio analysis result. These results are inside the frontiers of the respective flexible mating ratios by some distance. The extra value added by moving to flexible mating ratios can then be exploited as extra genetic gain, extra genetic diversity, or a combination of both, as illustrated by the red arc in Fig. 1 and the distance between the frontier curve A and B from C in Fig. 2. Results show that a flexible mating ratio for both sexes (curve A) gives considerable opportunity to increase selection response at the same level of coancestry/inbreeding rate. Moreover, this policy also provides good opportunity to reduce the inbreeding rate at a chosen level of predicted selection response. A flexible mating ratio for males alone has a less dramatic effect. The flexible mating structures for only males or for both males and females resulted in, respectively, 3.8 and 20.8% extra genetic gain for the same level of mean parental coancestry as achieved at 30^o^ under the fixed ratio scenario (Table 1).

## Population differentiation to supply markets with different target egg weights

An additional scenario was run to evaluate the opportunity to create divergent subpopulations based on a specific trait and to determine whether parents for divergent market needs could be sourced from the same population. Egg weight in period 1 (EW1) was selected as the target, as classification and pricing of table eggs based on size differs between countries and, therefore, the optimal egg weight is not the same across the globe. The index used for selection had a small weight on increasing EW1. As we are targeting production of progeny from current matings, this operates one generation ahead of dissemination to different uses or markets. This is because the pool of individuals to be divergently disseminated was planned for this purpose at mating in the previous generation. In the approach used, bimodality in the target trait is generated not only by selection but also by mate allocation.

When a default weighting of 1.0 was applied to diverging EW1, as illustrated by [16], progeny subpopulations that differed by 3.2 g (approximately 1.5 genetic standard deviations) in mean EBV for EW1 were obtained. This result came with no detriment to mean response in the index or in any trait, or in parental coancestry, because the weighting on diverging EW1 was not sufficient to affect animal selection. However, the resulting rearrangements in mate allocations gave an increase in progeny inbreeding from 0.005 to 0.006. By altering weightings on EW1 divergence vs. inbreeding control, a greater deviation in EW1 could be achieved at greater expense on progeny inbreeding [9]. This is a choice for the practitioner to make.

In this simple single-trait scenario, we are essentially practicing assortative mating on EW1, however it is possible to carry this out across multiple traits and/or genetic markers for two or more targeted end uses [17]. However, this trait-by-trait assortative mating approach is somewhat limited when multiple traits are targeted because, for example, matings that give high values for one trait are likely not the matings that also give high (or low) values for another trait. This can be addressed by specifying a different multi-trait index for each market, region, or another target outcome, and by practicing assortative mating on these indices [18]. This can be extended to additionally manage genetic markers and genetic diversity for each target market [17].

### Impact of imposing desired gains to maintain feed efficiency

In practice, individual traits may require additional constraints because direct application of the index can lead to undesirable responses in some traits. In our case, additional constraints may be recommended for traits related to efficiency (RFI, AFC, BW), delay in sexual maturity (AFE), and loss of YW%, as the response in the index was mostly driven by GEGG, BS, and EW.

**Table 2:** Predicted responses to selection for the index and individual traits

Trait	Mean of selection candidates	Reference	EW bimodal	EW bimodal + AFC < 0
Index	2.234	1.630	1.630 (100%)	1.586 (97.3%)
Average progeny inbreeding		0.005	0.006 (121.3%)	0.005 (114.9%)
AFC	0.060	0.850	0.847 (99.6%)	−0.064 (−7.5%)
AFE	0.710	0.566	0.578 (102.1%)	0.664 (117.3%)
BSE1	199.590	131.907	131.290 (99.5%)	126.638 (96%)
BSE3	172.593	126.452	126.510 (100%)	121.252 (95.9%)
BSPO1	182.636	195.923	196.514 (100.3%)	199.926 (102%)
BSPO2	180.890	201.407	202.275 (100.4%)	206.345 (102.5%)
BW	2.328	13.531	13.263 (98%)	−0.184 (−1.4%)
EM	1.089	0.809	0.807 (99.8%)	0.620 (76.6%)
EW1	0.761	0.386	0.381 (98.7%)	0.203 (52.6%)
EW2	0.854	0.405	0.401 (99%)	0.250 (61.7%)
EW3	0.849	0.438	0.435 (99.3%)	0.302 (68.9%)
GEGG1	0.808	0.181	0.180 (99.4%)	0.191 (105.5%)
GEGG2	2.201	1.588	1.593 (100.3%)	1.605 (101.1%)
GEGG3	3.202	2.475	2.479 (100.2%)	2.477 (100.1%)
GEGG4	3.340	2.822	2.827 (100.2%)	2.932 (103.9%)
RFI	−0.418	0.289	0.293 (101.4%)	−0.273 (−94.5%)
YW1	−0.003	0.001	0.001 (100%)	−0.072 (−7200%)
YWPT1	−0.346	−0.172	−0.169 (98.3%)	−0.214 (124.4%)

The responses are presented for the reference scenario, with a bimodal target for egg weight (EW1), and with a bimodal target for egg weight (EW) plus desired gain in average feed consumption (AFC) set < = 0. Values in brackets represent percent response relative to the Reference scenario

Table 2 shows expected responses in individual traits for the fixed mating ratio scenario, which shows an undesired increase of 0.850 g/d in feed intake (AFC) and of 0.289 g/d in residual feed intake (RFI), suggesting loss of feed efficiency. These increases in feed intake were more than compensated for by the increases in production, thereby maximizing genetic gain profit from selection on an index that is based on profit. However, this may be unacceptable for customers in regions with high feed prices or strong consideration for environmental impact. Therefore, using the tactical desired gains method described by [16], a scenario was run for the 30^o^ option with the same index but with desired target mean EBV for AFC of less than 0 g/d, just below the current mean EBV of selection candidates of 0.060 g/d. Thus, progeny mean EBV for AFC less than 0 g/d are acceptable and not penalized in any way, but solutions above 0 g are penalized. As the outcome depends on EBVs among the selection candidates, the target, in this case 0 g/d, is rarely exactly met. This constraint was applied in addition to targeting bimodality in EW1, as described in the previous section. This reflects real application, as it is quite typical for practitioners to apply multiple such constraints to traits, as well as on genetic markers, while managing compromises in overall index, coancestry, and other factors.

**Fig. 3:**
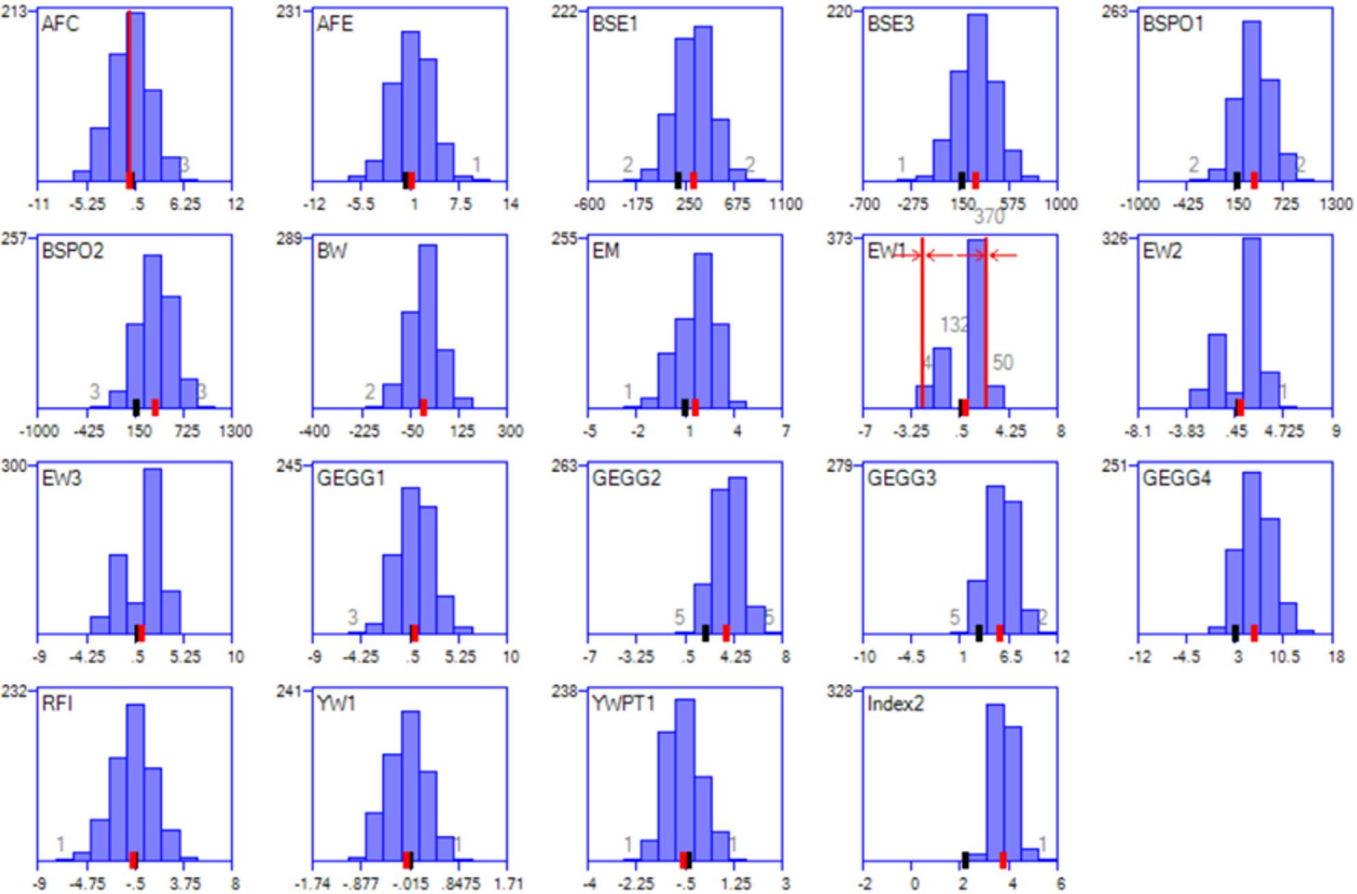
Response to selection in individual traits, when targeting bimodality in egg weight. Histograms of progeny mean EBVs (target mode means are the two vertical red lines on the EW1 histogram), with 70% of matings targeted to be in the upper mode, under a fixed mating ratio and a 30^o^ target. In addition, a constraint was applied for progeny mean EBV for average feed consumption (AFC) to be less than or equal to 0 (the red vertical line on the AFC histogram). In the graphs, the black ticks denote the mean EBV of the selection candidates, while red ticks denote the mean EBV of the selected individuals. The traits on the individual histograms are: saleable egg production rate in 4 time periods (GEGG1-4), average feed consumption (AFC), residual feed intake (RFI), egg mass (EM), egg weight (EW), breaking strength at equator (BSE) and poles (BSPO), yolk weight (YW), and yolk weight%

It is interesting to note that the divergence between the EW groups, as illustrated in Fig. 3, increased from 3.22 to 3.34 g when the AFC constraint was added. Moreover, progeny inbreeding was slightly reduced from 0.006 to 0.005. These outcomes are possible because the AFC constraint resulted in a different animal selection outcome, and this new selection was more conducive to accomplishing divergence in EW and lower progeny inbreeding. Results in Table 2 show that control of changes in AFC was achieved at a cost of a small reduction in EW gains. However, with greater improvements in shell strength, the response in the index was only 2.7% (0.044 units) lower than in the scenario without any trait management. All three scenarios in Table 2 gave the same control of mean parental coancestry (0.014), but some differences in average progeny inbreeding: 0.005, 0.006, and 0.005 for, respectively, no constraints, bimodal EW, and bimodal EW plus AFC constraint.

## Pooled semen and group mating

**Fig. 4:**
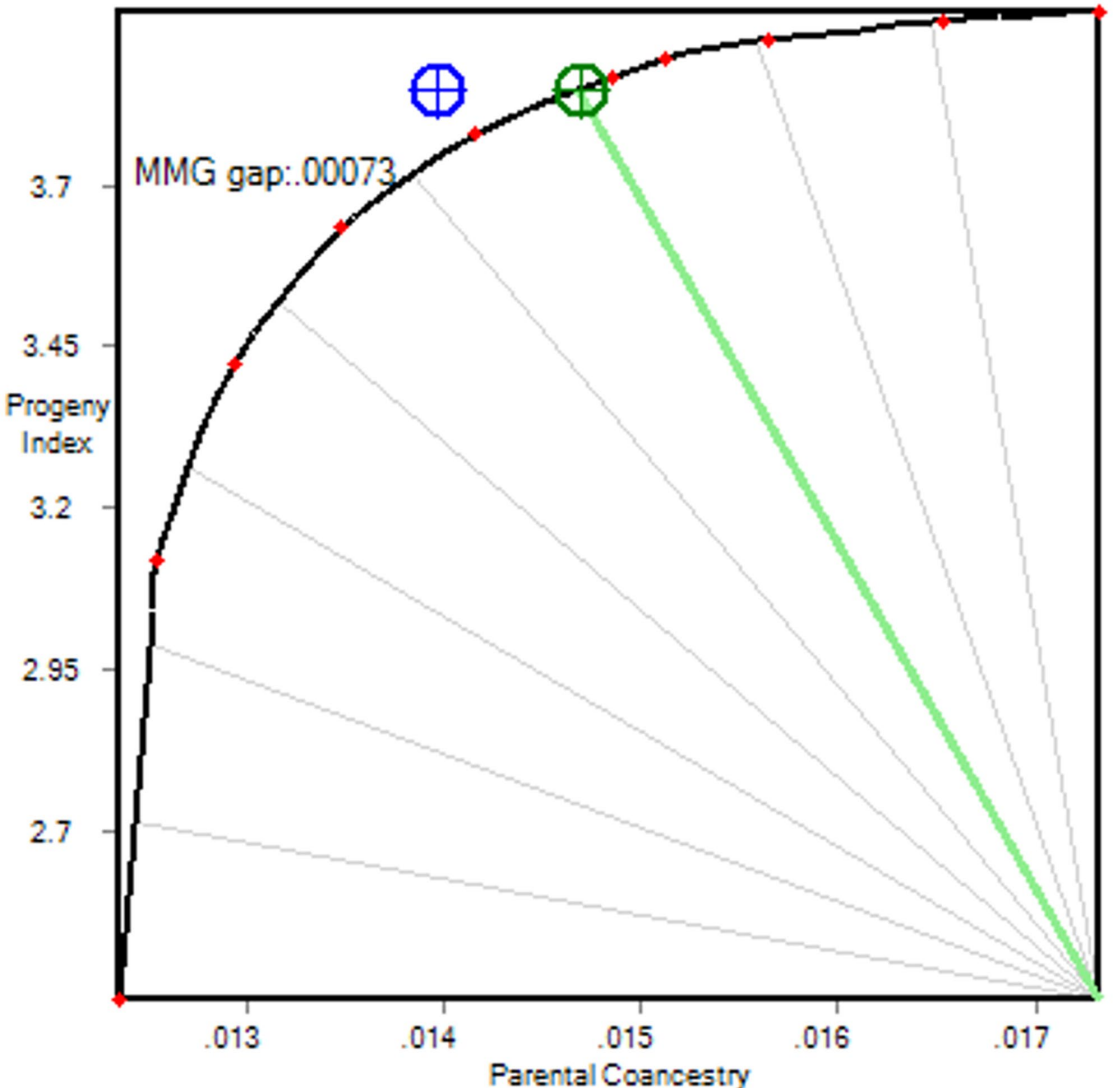
Impact of unequal male contributions under group mating. The curved line is the gain-diversity frontier, representing the maximum mean progeny index (vertical axis) that can be achieved for a given level of mean parental coancestry (horizontal axis). The average coancestries on this line have been corrected for variation of reproductive success among males in a pool. The circles indicate the optimal solutions at 30^o^. The blue solution naïvely assumes that all males within each mixed mating group (MMG) have equal mating success, leading to a 0.00073 lower average parental coancestry than predicted after correction. The green solution corrects for variation in male reproductive success. The algorithm reduces the gap between these solutions by increasing the mean relationship between males within groups while reducing mean relationships/coancestry across all groups

Another important practical aspect for poultry breeders is the use of pooled semen or larger floor mating pens. Pooled semen tends to increase fertility and female reproductive success, as there is variation in semen motility and fertilizing ability between roosters [19], which can result in the loss of contributions to the next generation of hens that are mated only to roosters with lower fertility and lower than desired progeny numbers from those roosters. For this analysis, instead of individual assignment of 100 males to 600 females, we created 10 groups, each containing 10 males and 60 females, using the mixed mating groups method described by [14]. This method divides males and females into groups with low within-group relationships between the genders to minimize inbreeding of progeny, unless other objective function components are overriding. If we assume that each male has equal success in fertilizing eggs, the suboptimal blue solution of Fig. 4 would result. However, if there is variation in male reproductive success within the pools of semen, a correction to predicted parental coancestry must be applied based on a prediction of this variation in male reproductive success. This correction results in the green-coloured solution of Fig. 4, which sits on the optimal frontier, resulting in lower retention of genetic diversity (or higher mean parental coancestry) than under the assumption of equal reproductive success. Of course, even without pooling, but using individual mate allocations, realized contributions to the next generation can also differ from what is planned in the mating list employed. However, the impact of this is likely to be much smaller than under the pooled semen scenario, largely because we have some control over mating. When using pooled semen, we lose that control, similar to using multiple males in a natural mating group. This issue is largely confined to males, as females generally do not have to compete with each other to get fertilised.

At 30^o^ under group mating, the mean response in progeny index (1.621) was similar to that of the single mating scenario (1.630) (Tables 1 and 2). As expected, the most aggressive policy (0^o^) gave the same predicted mean index result for both these scenarios, as illustrated by the maximum heights of curves C and D in Fig. 2 being equal, as the best 100 males and 600 females are used in both cases. However, the group mating strategy’s relative performance reduced as the policy became more conservative, because male reproductive success cannot be controlled in a competitive system, resulting in an increase in the mean parental coancestry.

**Fig. 5:**
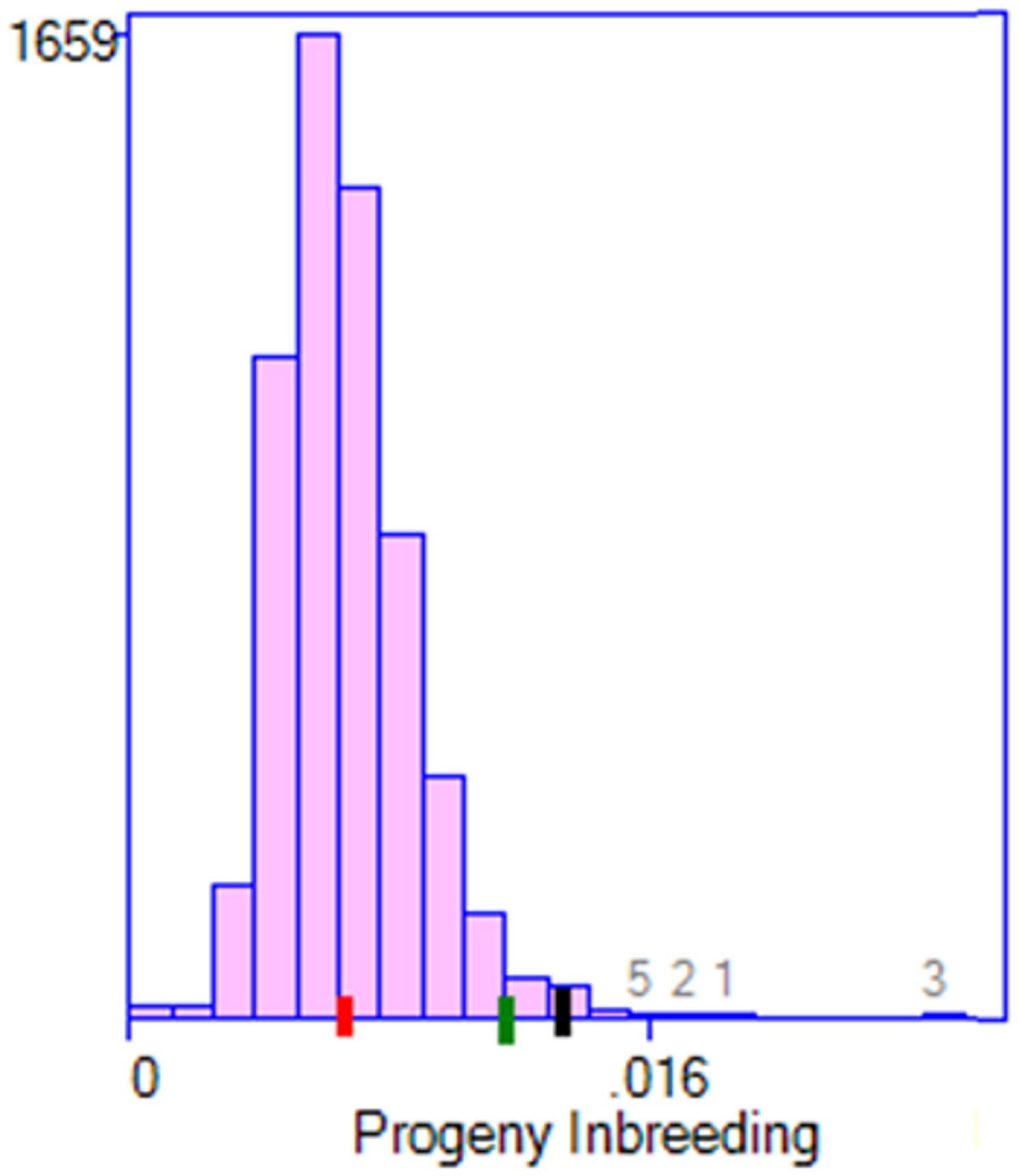
Distribution of progeny inbreeding with pooled semen and group mating. Histogram shows distribution covering all 600 possible matings within each of the 10 groups. The black tick is the expected mean progeny inbreeding with random mating among all *selection* candidates, the red tick is the expected mean inbreeding under the optimised solution, and the green tick is the expected mean inbreeding predicted from random mating among the *selected* candidates. This illustrates that most reduction in progeny inbreeding is from the mate allocation component, rather than from optimal contribution selection

The distribution of progeny inbreeding under group mating with varying fertility is shown in Fig. 5. The overall reduction in inbreeding (the difference between the red tick and the black tick) is made up of two components: that due to selection (the difference between the green tick and the black tick) and that due to mate allocation (the difference between the red tick and the green tick). Mean progeny inbreeding was higher with group mating than with individually assigned matings, but still low: 0.007, versus 0.005 (Table 1). This outcome is as expected, as all of the 600 possible matings within each mating group need to be considered, as there is no control over individual matings within a mating group, forgoing the possibility to choose specific matings that give very low progeny inbreeding. However, longer term inbreeding is more important than progeny inbreeding and this is controlled by the mean parental coancestry across all groups, which was still well controlled (Achieved Coancestry: 0.015 compared to 0.014 with individual matings).

With correction of average coancestries for variation in male reproductive success the grouping of males that are more closely related to each other is promoted, such that variation in fertilization success is less important. Moreover, aiming for low progeny inbreeding within groups also promotes closer relationships among males within groups, because, for a given group of females, the males that result in lower progeny inbreeding will tend to be genetically similar to each other. As expected, the mean coancestry within male groups (0.032) was higher than the mean coancestry across all male groups (0.014), but not as high as it could have been, because that would have increased the population mean parental coancestry too much. It is all a question of balance, which is achieved by applying an objective function that includes control of overall parental coancestry across all mating groups.

**Table 3:** Mean response in index, traits, and inbreeding with pooled semen and group mating

Trait	Mean	SD	Min	Max
Index	1.621 (99.4%)	0.110	1.465	1.835
AFC	0.881 (103.7%)	0.544	−0.006	2.021
AFE	0.621 (109.9%)	0.707	−0.676	1.706
BSE1	127.951 (97%)	47.070	32.045	183.590
BSE3	122.567 (96.9%)	39.126	68.339	174.545
BSPO1	193.885 (99%)	51.968	105.470	260.084
BSPO2	199.414 (99%)	58.833	83.344	262.461
BW	12.454 (92%)	12.716	−3.759	30.873
EM	0.826 (102.1%)	0.315	0.289	1.385
EW1	0.391 (101.2%)	0.360	−0.300	1.047
EW2	0.403 (99.6%)	0.387	−0.366	0.967
EW3	0.433 (98.9%)	0.469	−0.441	1.191
GEGG1	0.139 (76.7%)	0.457	−0.836	0.825
GEGG2	1.597 (100.6%)	0.273	1.176	2.055
GEGG3	2.472 (99.9%)	0.485	1.823	3.433
GEGG4	2.795 (99%)	0.942	1.204	4.431
RFI	0.332 (114.6%)	0.330	−0.100	1.082
YW1	0.003 (280%)	0.099	−0.117	0.200
YWPT1	−0.170 (99.3%)	0.188	−0.494	0.160
Progeny inbreeding	0.032 (687.2%)	0.012	0.013	0.056
Average coancestry of males	0.033	0.012	0.013	0.056

Mean response is included together with associated standard deviations, minima, and maxima across the 10 assigned mating groups. Values in brackets represent the percentage response relative to individual selection and mating assignment

Within groups, similar predicted response to selection was achieved in each group for the index (Table 3) but responses for individual traits varied considerably, with some groups compensating for lower gain in egg weight by better shell quality. Such differences between groups can also be intentionally targeted with use of different trait weightings for the different groups.

## Conclusions

The selection and mating strategies with control on inbreeding and trait outcomes that were pursued here (OCS combined with mating, flexible mating, group mating with variable reproductive efficiency, and targeting different markets) can be implemented in ongoing breeding programs and impact genetic gain and preservation of genetic diversity. With good effective population size, the inbreeding rate in the laying hen population evaluated here was low and could not be substantially reduced without significant loss in genetic gain or by resorting to flexible mating ratios. Flexible mating ratios provide an opportunity for additional gain and/or improved diversity management, however practical logistics of these selection and mating approaches would have to be critically evaluated. If the goal is to create divergent subpopulations to serve different markets, substantial differences can be created, e.g. in egg weight, with little or no impact on other economically important traits or genetic diversity. By applying tactical desired gains in individual traits, such as feed efficiency, based on prevailing EBVs, similar responses can be achieved for the index. Although we only applied two additional components to the objective function, practitioners often apply many such constraints to trait and genetic marker outcomes, while managing their impact on gains and diversity. In these cases, the impact on response in the index may be greater and will need careful evaluation. Mating groups can be optimally assigned for pooled semen or multi-sire floor mating situations without much compromise in overall selection response and coancestry compared to individual mating assignments, but at the expense of some loss of progeny inbreeding control. This optimization must account for the impact of variation in fertility between males on genetic diversity. Investigation of long-term effects of applying the discussed selection and mating strategies is important but was not undertaken with the real data analysis that was used here. However, this can be achieved with multi-generation simulation of alternative scenarios.

## Data Availability

The data that support the findings of this study are available from Hy-Line International, but restrictions apply to the availability of these data, which were used under license for the current study, and so are not publicly available. Data are however available from the authors upon reasonable request and with permission of Hy-Line International.
